# Proximal Anastomosis for the Treatment of Acute Type A Aortic Dissection: An Operative Technical Note

**DOI:** 10.3390/jcm15187156

**Published:** 2026-09-15

**Authors:** Marco Follis, Tomasz Urbanowicz, Antonio Segreto, Calogera Pisano, Marco Morsolini, Mariusz Kowalewski, Diego Bellavia, Giuseppe Maria Raffa, Fabrizio Follis

**Affiliations:** 1 Department of Cardio-Thoracic and Vascular Surgery, Klinikum Braunschweig, 38126 Braunschweig, Germany; 2 Cardio-Thoracic Surgery Department, Heart and Vascular Centre, Maastricht University Medical Centre, Cardiovascular Research Institute Maastricht (CARIM), 6229 HX Maastricht, The Netherlands; 3 Cardiac Surgery and Transplantology Department, Poznan University of Medical Sciences, ½ Długa Street, 61848 Poznan, Poland; 4 Cardiac Surgery, Department of Precision Medicine in Medical Surgical and Critical Area (Me.Pre.C.C.), University of Palermo, 90134 Palermo, Italy; 5 Mediterranean Institute for Transplantation and Advanced Specialized Therapies (IRCCS ISMETT), 90127 Palermo, Italyffollis@ismett.edu (F.F.); 6 Thoracic Research Centre, Innovative Medical Forum, Collegium Medicum in Bydgoszcz, Nicolaus Copernicus University, 85067 Bydgoszcz, Poland; 7 Department of Cardiac Surgery and Transplantology, National Medical Institute of the Ministry of Interior, 02507 Warsaw, Poland

**Keywords:** acute type A aortic dissection, proximal anastomosis, proximal graft invagination technique, graft invagination, aortic surgery, sinotubular junction

## Abstract

This Technical Note describes a proximal anastomotic technique for supracoronary ascending aortic replacement in acute type A aortic dissection. The method is based on invagination of a short prosthetic graft segment at the sinotubular junction, followed by eversion to create a reinforced proximal cuff. The technique aims to facilitate restoration of aortic valve coaptation, reduce proximal suture-line tension, and improve intraoperative hemostasis in fragile or dissected aortic tissue. The same principle may also be considered in selected elective aortic procedures when reinforcement of the proximal anastomosis is desirable.

## 1. Introduction

Acute type A aortic dissection (ATAAD) is a life-threatening condition requiring immediate surgical intervention. Despite advances in perioperative care and surgical technique, mortality remains substantial. Large surgical series report 30-day mortality rates ranging from 8.9% to 23.9%, with more recent data from high-volume registries such as the German Registry for Acute Aortic Dissection Type A (GERAADA) and the International Registry of Acute Aortic Dissection (IRAD) showing operative mortality between 8.9% and 16.9% [1,2]. Early mortality within 48 h remains notable, at 4.4% among surgically treated patients and 5.8% among all patients presenting with ATAAD [2]. Overall in-hospital mortality is also substantial, with reports up to 22% [3].

In addition to early survival, the quality and durability of the proximal reconstruction remain central technical concerns. The dissected proximal aortic wall is often fragile, edematous, and prone to bleeding, making a tension-free and hemostatic suture line difficult to obtain. Late pseudoaneurysm formation after proximal repair represents an additional concern and has been cited as an important cause of morbidity and reoperation in longitudinal series [4]. Therefore, operative strategies that reinforce the proximal anastomosis while preserving valve function are clinically relevant.

The proximal suture line is also the point at which several competing surgical priorities converge: rapid restoration of aortic continuity, reliable sealing of the dissected media, preservation or restoration of aortic valve competence, and avoidance of excessive manipulation of the aortic root. Conventional reinforcement methods, including felt sandwich techniques, adventitial inversion or eversion, prosthesis eversion, and turn-up configurations, were developed to address these priorities with different balances between speed, hemostatic security, and technical complexity [5,6,7,8,9,10,11,12,13]. However, no single method can be considered universally optimal, because the proximal stump may vary widely according to the extension of the intimal tear, the degree of root involvement, the presence of aortic regurgitation, and the quality of the residual adventitial layer. A Technical Note is therefore useful when it defines a reproducible operative concept, specifies the anatomical circumstances in which the method is appropriate, and clarifies the technical details that reduce the risk of bleeding or valve distortion.

We describe a modified application of the classical “elephant trunk” principle, referred to here as the “proximal graft invagination technique”, applied exclusively at the proximal anastomosis. Unlike conventional elephant trunk approaches directed toward distal aortic pathology, this technique uses a short prosthetic graft segment that is invaginated within the aortic root at the sinotubular junction (STJ) and subsequently everted outward to create a reinforced proximal cuff.

The rationale of the technique is to provide a stable prosthetic interface for suturing, reduce mechanical stress on dissected native tissue, and facilitate valve-sparing supracoronary replacement when the aortic leaflets are suitable for preservation. Although the method introduces an additional graft-to-graft anastomosis, modern Dacron vascular prostheses provide high mechanical integrity and predictable handling characteristics.

In this Technical Note, we detail the operative steps, clarify key technical points and potential pitfalls, and discuss the advantages and limitations of the technique in the context of previously described proximal reinforcement strategies.

## 2. Materials and Methods

Intraoperative transesophageal echocardiography is essential before repair to assess the STJ, aortic root, and aortic annulus, and to evaluate aortic leaflet morphology under dynamic conditions. Root replacement (prosthetic or valve-sparing) is required when there is a marked leaflet abnormality, inadequate coaptation, or aortic regurgitation of uncertain mechanism, causing acute heart failure, or decompensation of pre-existing, chronic regurgitation, and when the regurgitation cannot be corrected by commissural resuspension.

After median sternotomy, cardiopulmonary bypass is established through right axillary or femoral arterial cannulation according to the clinical scenario and institutional preference and double venous cannulation. The patient is cooled to 28 °C (25 °C if arch replacement is needed), the head is packed in ice, the aorta is cross-clamped and cardioplegia is delivered into the coronary ostia after resection of the dissected ascending aorta. The ascending aorta is transected proximally at the level of the STJ. The layers are reapproximated and the false lumen is obliterated using surgical adhesive or multiple pledget-supported sutures. Commissural resuspension is then performed to stabilize the aortic valve, which in the majority of patients with ATAAD restores valve competency without root replacement [14].

**Figure 1:**
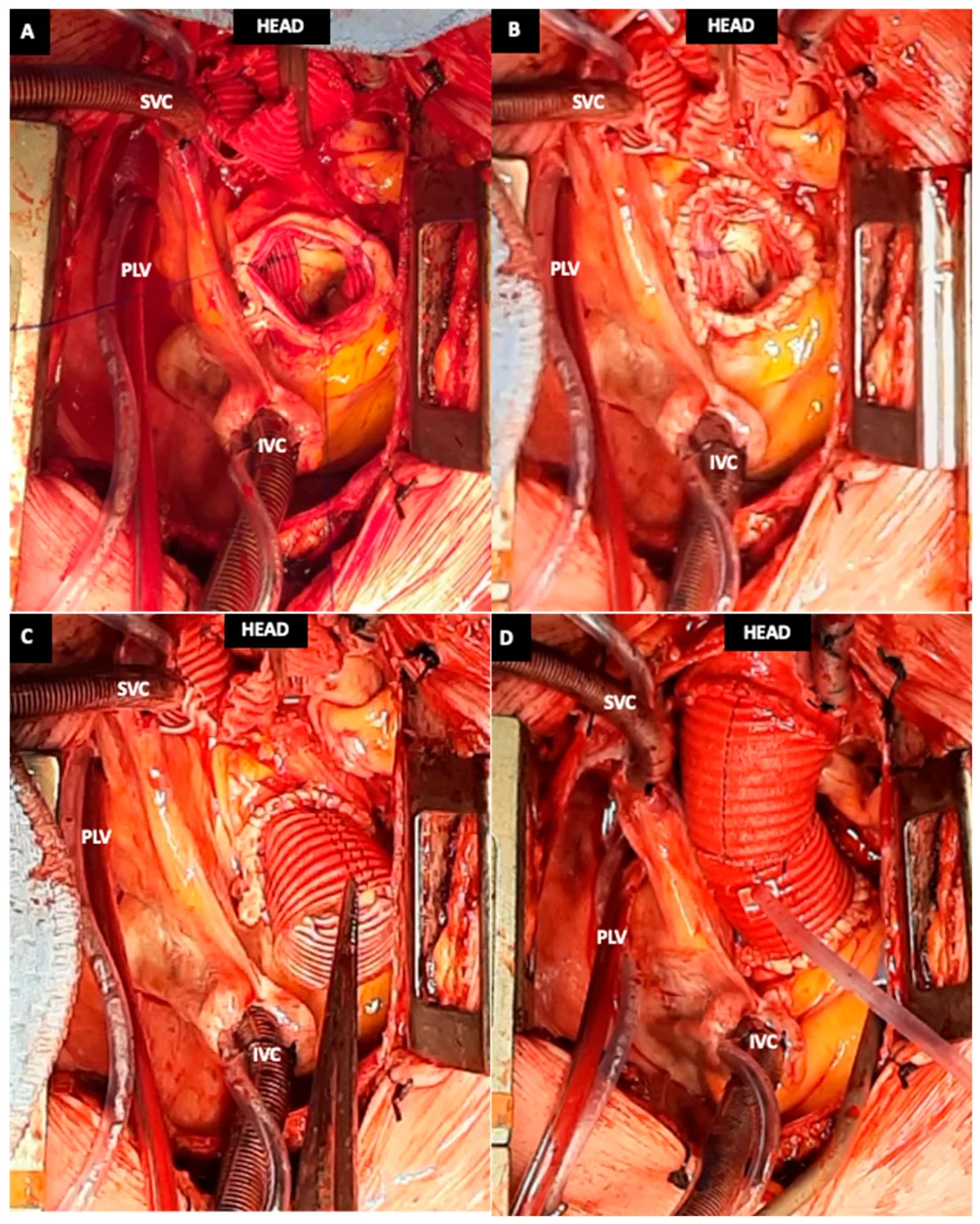
Proximal graft invagination technique for treatment of acute type A aortic dissection. (**A**) Vascular graft invaginated into the sinotubular junction; distal vascular graft anastomosed and clamped. SVC: superior vena cava cannulation; IVC: inferior vena cava cannulation; PLV: pulmonary vein venting. (**B**) Proximal anastomosis with the invaginated vascular graft. (**C**) The prosthesis is everted. (**D**) Completion of the end-to-end graft-to-graft anastomosis.

After adequate hypothermic cooling, circulatory arrest is initiated with selective antegrade cerebral perfusion through the axillary cannula and retrograde cerebral perfusion via the cannula in the superior vena cava in case of femoral cannulation. The distal anastomosis to the aortic arch is completed using standard techniques; antegrade systemic perfusion is resumed, and the prosthesis is clamped. During rewarming, attention is directed to the aortic root: the STJ is measured to select a prosthetic graft size that supports the native geometry without distortion or mismatch. A 2.5 cm segment of the selected dacron prosthesis is cut and invaginated into the proximal aortic stump (Figure 1A) and fixed at each commissure using three interrupted 4-0 polypropylene sutures (Figure 1B). When the native tissue is particularly fragile, an external and internal (double polytetrafluoroethylene (PTFE) strip technique) PTFE felt reinforcement may be added to improve anastomotic strength [5]. The invaginated graft segment is then everted outward (Figure 1C), creating a stable reinforced cuff for the proximal anastomosis. The repair is finalized with a graft-to-graft anastomosis using a 5-0 polypropylene running suture (Figure 1D).

Several technical points are important for reproducibility. First, the graft should be selected according to the measured STJ rather than intentionally oversize it, because excessive prosthetic support may distort the commissural geometry and impair leaflet coaptation. Second, commissural fixation should be symmetrical and performed before final eversion of the graft segment, so that the native commissures are stabilized in their physiological orientation. Third, the invaginated portion (2.5 cm) should remain limited to the proximal cuff and should not be advanced across the aortic valve plane. This distinguishes the technique from transvalvular prosthesis invagination and is intended to reduce the risk of leaflet abrasion, subannular trauma, and interference with valve motion. Fourth, after eversion, the proximal cuff should be inspected circumferentially, with particular attention to the commissural regions and to any area where the dissected layers remain mobile. Additional pledgeted stitches or external felt may be used selectively when the native tissue does not provide sufficient support.

The technique is best suited to cases in which the primary objective is supracoronary ascending aortic replacement with preservation of a competent or repairable native aortic valve. It should be used cautiously when the STJ is very small, when the intimal tear extends deeply into the root, when cusp disease is responsible for relevant aortic regurgitation, or when the root anatomy requires a formal root replacement or valve-sparing root procedure [5]. Intraoperative transesophageal echocardiography after weaning from cardiopulmonary bypass is therefore essential to confirm valve competence, exclude residual significant regurgitation, and assess the geometry of the reconstructed proximal aorta.

## 3. Results

The intended technical result is a reinforced, hemostatic, and geometrically stable proximal anastomosis at the STJ. By interposing an invaginated and subsequently everted prosthetic cuff, the suture line is created between closely opposed prosthetic and native surfaces, with reduced tension on the dissected aortic wall. This configuration also allows valve-sparing supracoronary replacement when intraoperative echocardiography and direct inspection confirm adequate leaflet morphology and correctable commissural alignment.

In our initial experience at Ismett-UPMC, the technique was associated with satisfactory intraoperative hemostasis and no early proximal anastomotic failure. In the case series of six patients in which this technique has been adopted, no proximal anastomotic pseudoaneurysm has been detected at one-year follow-up computed tomography (CT) scan. These observations should be interpreted as technical feasibility and early imaging follow-up findings rather than evidence of comparative clinical superiority, because the present report is not designed as a controlled outcome study.

From a technical standpoint, the most relevant intraoperative endpoint is the achievement of a dry proximal suture line after systemic reperfusion and reversal of anticoagulation, without the need for extensive additional root manipulation. The postoperative imaging endpoint is the absence of a focal contrast-filled outpouching or progressive enlargement at the proximal anastomotic level on follow-up CT. In the present institutional experience, the available follow-up CT scans did not show proximal pseudoaneurysm formation. Because the number of treated patients and the follow-up duration are limited, these findings are presented descriptively and are intended to support the feasibility of the technique rather than to establish its long-term superiority.

## 4. Discussion

The proximal anastomosis in ATAAD remains technically demanding because of the fragility of dissected aortic tissue and the need for rapid, hemostatic, and reproducible reconstruction. Several reinforcement strategies have been proposed to address these challenges. Tsutsui et al. described a modified turn-up technique in which a short graft collar is rolled back and secured to an external PTFE strip to simplify needle passage and improve stability, particularly for early-career surgeons [5]. Rignano et al. described a prosthesis eversion technique in which an inverted Dacron graft is advanced across the aortic valve into the left ventricle, then everted and sutured to the annulus [6]. More recently, adventitial eversion reinforced with an autologous pericardial strip has also been reported [7].

The growing number of proximal reinforcement techniques in the literature reflects the absence of a universally accepted solution for this specific surgical problem. Tamura et al. reported the original turn-up concept, in which eversion of the graft ends improves cross-sectional exposure of the aortic wall and prosthesis, thereby facilitating completion of the running suture and identification of bleeding points [8]. Later clinical experience with turn-up anastomosis suggested that this principle can provide stable hemostasis in larger ATAAD series [12]. Similarly, comparative studies of prosthesis eversion and adventitial eversion strategies have emphasized that better apposition between the prosthesis and the dissected aortic stump may reduce bleeding and improve early outcomes [9,10]. More recent technical reports, including the reversed turn-up method, have focused on the posterior proximal wall, where bleeding may be particularly difficult to access once the heart is filled and systemic pressure is restored [11]. These studies collectively support the concept that the geometry of the proximal suture line is as important as the suture material itself.

The proximal invagination technique differs from these approaches in several practical aspects. It avoids transvalvular passage of the graft, thereby limiting potential contact with the aortic cusps and subannular structures. It also avoids dependence on extensive adventitial eversion, which may be difficult in fragile or severely dissected tissue. The short invaginated graft segment provides an internal prosthetic scaffold at the STJ and, after eversion, creates a reinforced proximal cuff that can be completed with familiar suturing principles. If tissue quality is poor, external and internal PTFE felt can still be added, preserving versatility without making it mandatory.

Within this context, the proposed technique can be interpreted as a proximal cuff strategy rather than a root replacement strategy. The invaginated graft segment creates an internal landing zone that distributes tension over a broader surface, while eversion converts this internal support into an accessible external cuff. This sequence may be particularly useful in emergency ATAAD repair, where the surgeon must obtain hemostasis quickly but also avoid excessive root dissection. Compared with techniques that require extensive adventitial mobilization, the proximal invagination approach relies primarily on prosthetic geometry and commissural anchoring. Compared with techniques that pass the graft through the valve, it maintains the prosthesis above the valve plane. The technique therefore aims to combine the hemostatic logic of eversion-based repairs with a valve-sparing, STJ-limited application.

This report should be interpreted within its limitations. Although hemostasis was adequate, the postoperative course was uneventful and no early proximal anastomotic failure or pseudoaneurysm was observed at one-year follow-up, the experience is preliminary, the number of treated patients is small (*n* = 6) and there is no control group or mature, standardized long-term imaging follow-up. Therefore, no definitive conclusion can be drawn regarding superiority over other proximal reinforcement methods. Potential drawbacks include the additional graft-to-graft interface, which could theoretically become a bleeding site, and technical difficulty in very small STJs (<26 mm). Nevertheless, because pseudoaneurysm formation after ATAAD repair has been reported at rates ranging from 10% to 24% [15,16], and because pseudoaneurysm remains a relevant cause of late reintervention [17], techniques aimed at improving proximal suture-line durability deserve continued evaluation. Prospective comparative studies with standardized imaging and clinical follow-up are required to determine whether this approach provides durable advantages.

Another relevant limitation is that the present manuscript does not quantify operative time, transfusion requirements, postoperative drainage, or the exact duration of imaging follow-up. For this reason, the absence of pseudoaneurysm on the latest available CT scans should be considered an early safety observation. Future studies should prospectively collect standardized operative variables, early bleeding endpoints, valve competence on echocardiography, and serial CT measurements of the proximal anastomosis. Such a design would allow direct comparison with felt sandwich, adventitial inversion or eversion, prosthesis eversion, and turn-up techniques, and would determine whether the theoretical advantages of reduced suture-line tension and limited valve manipulation translate into measurable clinical benefit.

## 5. Conclusions

The proximal graft invagination technique is a reproducible graft invagination and eversion strategy for reinforcing the proximal anastomosis during supracoronary ascending aortic replacement in ATAAD. Its main technical objectives are improved hemostasis, reduced suture-line tension, preservation of aortic valve competence when anatomically appropriate, and simplified handling of fragile dissected tissue. These observations are preliminary; comparative effectiveness and long-term durability require prospective evaluation.

## Data Availability

No new data were created or analyzed in this study. Data sharing is not applicable to this article.

## Abbreviations

The following abbreviations are used in this manuscript:

ATAAD	Acute type A aortic dissection
GERAADA	German Registry for Acute Aortic Dissection Type A
IRAD	International Registry of Acute Aortic Dissection
STJ	Sinotubular junction
PTFE	Polythetrafluoroethylene
CT	Computed Tomography
